# Reduced Expression of TMEM16A Impairs Nitric Oxide-Dependent Cl^−^ Transport in Retinal Amacrine Cells

**DOI:** 10.3389/fncel.2022.937060

**Published:** 2022-07-27

**Authors:** Tyler Christopher Rodriguez, Li Zhong, Hailey Simpson, Evanna Gleason

**Affiliations:** Department of Biological Sciences, Louisiana State University, Baton Rouge, LA, United States

**Keywords:** retina, nitric oxide (NO), amacrine cell, TMEM16/anoctamin, CRISPR/Cas9, intracellular Cl^−^

## Abstract

Postsynaptic cytosolic Cl^−^ concentration determines whether GABAergic and glycinergic synapses are inhibitory or excitatory. We have shown that nitric oxide (NO) initiates the release of Cl^−^ from acidic internal stores into the cytosol of retinal amacrine cells (ACs) thereby elevating cytosolic Cl^−^. In addition, we found that cystic fibrosis transmembrane conductance regulator (CFTR) expression and Ca^2+^ elevations are necessary for the transient effects of NO on cytosolic Cl^−^ levels, but the mechanism remains to be elucidated. Here, we investigated the involvement of TMEM16A as a possible link between Ca^2+^ elevations and cytosolic Cl^−^ release. TMEM16A is a Ca^2+^-activated Cl^−^ channel that is functionally coupled with CFTR in epithelia. Both proteins are also expressed in neurons. Based on this and its Ca^2+^ dependence, we test the hypothesis that TMEM16A participates in the NO-dependent elevation in cytosolic Cl^−^ in ACs. Chick retina ACs express TMEM16A as shown by Western blot analysis, single-cell PCR, and immunocytochemistry. Electrophysiology experiments demonstrate that TMEM16A functions in amacrine cells. Pharmacological inhibition of TMEM16A with T16inh-AO1 reduces the NO-dependent Cl^−^ release as indicated by the diminished shift in the reversal potential of GABA_A_ receptor-mediated currents. We confirmed the involvement of TMEM16A in the NO-dependent Cl^−^ release using CRISPR/Cas9 knockdown of TMEM16A. Two different modalities targeting the gene for TMEM16A (*ANO1*) were tested in retinal amacrine cells: an all-in-one plasmid vector and crRNA/tracrRNA/Cas9 ribonucleoprotein. The all-in-one CRISPR/Cas9 modality did not change the expression of TMEM16A protein and produced no change in the response to NO. However, TMEM16A-specific crRNA/tracrRNA/Cas9 ribonucleoprotein effectively reduces both TMEM16A protein levels and the NO-dependent shift in the reversal potential of GABA-gated currents. These results show that TMEM16A plays a role in the NO-dependent Cl^−^ release from retinal ACs.

## Introduction

The flexibility of synaptic signaling determines the ability of a neural circuit to alter its performance under different demands. Over 40 subtypes of retinal ACs were identified in mouse (Hel mstaedter et al., 2013) with more than 60 subtypes discovered by transcriptome analysis (Yan et al., 2020). ACs subserve diverse functions, such as generating cholinergic waves during retinal development (Feller et al., 1996), regulating activity-dependent maturation of ganglion cell projections (Xu et al., 2016), shaping receptive fields of ganglion cells (Jacoby et al., 1996; de Vries et al., 2011; Kim et al., 2015; Jia et al., 2020), and light and dark adaptation of retinal circuitry (Hampson et al., 1992; Zhang et al., 2008; Ortuno-Lizaran et al., 2020). AC's extreme morphological and functional diversity increases circuit capabilities in the IPL.

Nitric oxide (NO) is a signaling molecule produced by multiple cell types in the chicken retina including four nitric oxide synthase expressing (NOS) AC subtypes (Fischer and Stell, 1999). Two different isoforms of nitric oxide synthase (nNOS and eNOS) were shown to be expressed in avian ACs as well (Tekmen-Clark and Gleason, 2013). Three AC types with nNOS immunoreactivity were found in turtle (Eldred and Blute, 2005), and single-cell RNA sequencing showed three AC types with nNOS expression in mouse (Yan et al., 2020). NO regulates multiple aspects of retinal physiology including phototransduction (Goldstein et al., 1996), retinal blood flow (Izumi et al., 2008), temporal response tuning (Vielma et al., 2014), gap junction coupling (Hampson et al., 1992; Mills and Massey, 1995; Jacoby et al., 2018), and light adaptation (Shi et al., 2020).

In previous work, we showed that postsynaptic cytosolic Cl^−^ concentration can be elevated by NO thus making GABA-mediated synaptic responses less inhibitory, or even depolarizing (Hoffpauir et al., 2006). We have also shown that this response requires the cystic fibrosis transmembrane conductance regulator (CFTR) (Krishnan et al., 2017). In separate studies, we showed that NO (Zhong and Gleason, 2021) and NO donors (Maddox and Gleason, 2017) produce Ca^2+^ elevations that are required for the effects of NO on cytosolic Cl^−^, raising the possibility that internal Ca^2+^-activated Cl^−^ channels also contribute to the NO-dependent release of Cl^−^.

The TMEM16 family of transmembrane proteins is exclusive to eukaryotes with vertebrates expressing 10 paralogues: TMEM16A, TMEM16B, TMEM16C, TMEM16D, TMEM16E, TMEM16F, TMEM16G, TMEM16H, TMEM16J, and TMEM16K (Milenkovic et al., 2010). Most are activated by Ca^2+^ and function as ion channels, lipid scramblases, or sometimes both (Charlesworth et al., 2012; Yang et al., 2012; Huang et al., 2013; Suzuki et al., 2013; Kim et al., 2018; Bushell et al., 2019; Feng et al., 2019; Reichhart et al., 2019; Kalienkova et al., 2021; Stabilini et al., 2021). TMEM16 proteins are expressed in many tissues and are involved in a range of physiological processes including nociception (Cho et al., 2012; Takayama et al., 2015), mucous secretion (Benedetto et al., 2019), olfactory transduction (Pifferi et al., 2012; Neureither et al., 2017; Zak et al., 2018), bone remodeling (Kim et al., 2019), muscle repair (Whitlock et al., 2018), and macrophage immune defense (Ousingsawat et al., 2015; Wanitchakool et al., 2017). TMEM16A was first identified as the native Ca^2+^ activated Cl^−^ channel in *Xenopus* oocytes (Yang et al., 2008). Later, it was found in both the outer and inner plexiform layers of the mammalian retina and in GABAergic amacrine cell processes (Jeon et al., 2013). TMEM16A function was demonstrated in photoreceptor synaptic terminals (Caputo et al., 2015) and bipolar cell synapses (Paik et al., 2020) but not in amacrine cells.

TMEM16A is reported to function as a heat sensor in rat nociceptive dorsal root ganglion neurons that is synergistically activated by heat, Ca^2+^, and depolarizing potentials (Cho et al., 2012). Intracellular sources of Ca^2+^, in particular the endoplasmic reticulum, were found to be preferred over voltage-gated Ca^2+^ channel-mediated Ca^2+^ influx in rat DRG neurons. The preference for intracellular Ca^2+^ stores likely coincides with close membrane juxtaposition of TMEM16A and molecular components of Ca^2+^ signaling in localized microdomains (Jin et al., 2013). It was shown that TMEM16A, TRPV1, and IP_3_Rs were organized in nanodomains that couple ER Ca^2+^ release to TMEM16A activation (Shah et al., 2020).

Functional interactions between TMEM16A and CFTR have been demonstrated in several epithelia. Studies performed in mouse airway epithelia showed a pronounced interdependence between TMEM16A and CFTR in terms of membrane expression and Cl^−^ conductance (Ruffin et al., 2013; Benedetto et al., 2017). To date, this functional interdependence has not been explored in neurons. CFTR is a protein kinase A-regulated Cl^−^ and HCO${}_{3}^{-}$ channel localized to the apical membrane of epithelial cells that functions to passively secrete Cl^−^ thereby regulating sodium transport, transepithelial water flow, and luminal pH (Hull, 2012; Saint-Criq and Gray, 2017). Although CFTR is widely expressed at the plasma membrane of epithelial cells, it is also found in non-epithelial tissues (Yoshimura et al., 1991; Xue et al., 2016; Reznikov, 2017) with subcellular localization differing among cell types (Bradbury, 1999). When expressed intracellularly, it regulates processes such as plasma membrane recycling (Bradbury et al., 1992), endosomal pH homeostasis (Lukacs et al., 1992), and Cl^−^ dependent endosome fusion (Biwersi et al., 1996). Mutations in the *CFTR* gene are responsible for cystic fibrosis leading to sticky and viscous mucous. TMEM16A may also contribute to mucous secretion; however, this is disputed (Simoes et al., 2019; Danahay et al., 2020; Cabrita et al., 2021). Because functional interactions between CFTR and TMEM16A are established in epithelia, we hypothesize that in ACs, TMEM16A provides a link between NO-induced Ca^2+^ elevations and Cl^−^ release in tandem with CFTR.

Here, we test for the involvement of TMEM16A in the NO-dependent cytosolic Cl^−^ release. The transcript for TMEM16A was identified in mixed retinal cultures and in single ACs. Protein expression was confirmed using Western blots and immunocytochemistry. With electrophysiological experiments designed to monitor cytosolic Cl^−^, we look at the effects of pharmacological inhibition of TMEM16A and genetic disruption of TMEM16A with CRISPR/Cas9. We find that inhibition or reduced expression of TMEM16A suppresses the cytosolic Cl^−^ elevation produced by NO. This is the first report of TMEM16A participating in Cl^−^ regulation in ACs.

## Methods

### Cell Culture

To obtain cultures of retinal cells for use in electrophysiology, immunocytochemistry, and RT-PCR experiments, retinae were extracted from E9 White Leghorn chick embryos in Hanks Balanced Salt Solution (HBSS), Ca^2+^, and Mg^2+^ free (Corning, 21-021-CV). Retinae were digested with 0.125% trypsin (Thermo Fisher, 25200-056), treated with DNase (Sigma, DN25-1G), and gently triturated to generate a single-cell suspension. The cells were resuspended in Dulbecco's Modified Eagle Medium (DMEM) (Corning, 15-017-CV) containing 5% fetal bovine serum (FBS) and plated at a density of 260cells/mm^2^ onto 35mm dishes coated with poly-L-ornithine (Alamanda Polymers, PLO_100_ average MW 20,000Da). Half of the media was replaced every 2–3d with Neurobasal (Thermo Fisher, 21103-049) containing 1% B27 (Thermo Fisher, 17504-044) and 2mM GlutaMAX (Thermo Fisher, 35050-061). ACs were identified in culture as previously described (Gleason et al., 1993).

### Mixed Population RT-PCR

Total RNA was extracted using the acid phenol/guanidinium isothiocyanate method (Chomczynski and Sacchi, 2006). The RNA was then digested by DNase I, and acid phenol/guanidinium isothiocyanate was extracted again before final resuspension in RNA storage buffer (1mM citrate pH 6.5). Reverse transcription of RNA was carried out as follows: primers were annealed at 75°C for 5min before the addition of WarmStart RTX (except for no RT control) and cDNA synthesized by incubating at 25°C for 5min, 55°C 10min, 80°C for 10min, and then holding at 4°C. PCR amplification of cDNA was performed with Q5 Hot Start High-Fidelity DNA polymerase. Cycling parameters are as follows: 98°C for 30s for initial denaturation, then 11 cycles of 98°C for 5s, 65°C for 20s (−1°C/cycle), and 72°C for 45s, followed by 26 cycles of 98°C for 5s, 55°C for 20s, and 72°C for 45s, with a final extension at 72°C for 5 min.

### Single-Cell RT-PCR

Retinal cultures were washed three times with Tris-buffered saline (Fisher Bioreagents, BP24711). Individual ACs (1cell/rxn) were aspirated with a siliconized borosilicate glass pipette pulled to a tip diameter of ~5 μm filled with Tris-buffered saline. The collection was performed on an Olympus IX-70 inverted microscope with a micromanipulator (Sutter instrument, MPC-385). The glass pipette tip, containing the AC, was broken into a 1.5ml tube on ice containing 4 μl of dsDNase mix with 0.2 μl dsDNase per reaction (Thermo Fisher, EN0771). DNA was digested at 37°C for 2min. Reverse transcription components (NEB, B0537S, M0314S, and N0446S) were added to a final volume of 15μl, and RNA was denatured at 75°C for 5min. The RecA protein from *Thermus thermophilus* (MCLAB, RPTT-100) was included during RNA denaturation to improve primer hybridization and subsequent cDNA synthesis (Kirkpatrick and Radding, 1992). WarmStart RTX (NEB, M0380S) was added to each sample, except no-reverse transcription controls, and cDNA synthesis was performed as before. The cDNA (2μl/rxn) was then amplified with Q5 Hot Start High-Fidelity DNA polymerase (NEB, M0493S). Touchdown cycling parameters were as follows: 98°C for 30 s for initial denaturation, then 11 cycles of 98°C for 5 s, 65°C for 20 s (−1°C/cycle), and 72°C for 45 s, followed by 41 cycles of 98°C for 5 s, 55°C for 20 s, and 72°C for 45 s, with a final extension at 72°C for 5 min. Reactions were analyzed on a 2% lithium borate agarose gel poststained with ethidium bromide, and sequence identity was verified by Sanger sequencing.

### Single-Cell Genomic PCR

Genomic PCR was performed using similar methods for single-cell RT-PCR with the following modifications. Ice-cold HBSS (minus Ca^2+^ and Mg^2+^) was used to fill the siliconized pipette and collect the cells. The pipette, containing a single AC, was broken into a PCR strip tube on ice containing 5 μl of freshly prepared lysis buffer which comprised of 1mM tris pH 9.0 (Millipore, 9295-OP), 0.1 mg/ml proteinase K (Thermo Fisher, 17916), and 0.01 mM EDTA (Sigma, 03677). The strip was incubated in a thermocycler preheated to 65°C for 2hr and 80°C for 20 min before proceeding to PCR amplification.

### Immunocytochemistry

To determine the cellular localization pattern of TMEM16A protein, we performed immunocytochemistry on cultured retinal cells. Mixed retinal cultures plated on coverslips were fixed after 9 days in culture with 4% paraformaldehyde (Thermo Fisher, AA433689L) in Dulbecco's PBS minus Ca^2+^ and Mg^2+^ (Corning, 20-031-CV) for 15 min at room temperature. Cells were washed three times with DPBS and then permeabilized 15 min with DPBS containing 0.1% Tween 80 (VWR, 97063-806) and 30mM glycine. The cells were blocked with 5% NGS before incubating for 24 h in primary anti-TMEM16A rabbit monoclonal antibody (Abcam, ab190803). Cells were incubated for 1 h with secondary antibody and mounted onto slides using ProLong Glass Antifade Mountant (Thermo Fisher, P36984). The cells were washed once, briefly, with permeabilization buffer followed by three times for 5 min each with DPBS after antibody incubation steps.

### Western Blot Analysis

To assess the target specificity of the rabbit anti-TMEM16A monoclonal antibody, we performed Western blots to compare the molecular weight of the detected protein with its predicted size. In addition, we compared the immunodetection of TMEM16A and CFTR from membrane extracts of two methods to assess the possibility they interact and to verify that the anti-TMEM16A antibody recognizes a transmembrane protein as expected. Retinal tissue was dissected from chicks on embryonic day 18. Ultracentrifuged samples were lysed with a syringe plunger (Norm-Ject, 4010.200V0) in a microcentrifuge tube. Samples were spun at 700x*g* for 5 min in a fixed-angle centrifuge. Supernatants were spun at 100,000x*g* for 1 h at 4°C in a swinging bucket rotor to pellet the microsomal fraction. The pellet was resuspended in HEPES buffer with 0.5% CHAPS (VWR, 97061-720) and both fractions (supernatant and pellet) were then methanol-precipitated to concentrate and delipidate proteins. Triton X-114 cloud point extraction was performed in a separate experiment to partition hydrophobic proteins from soluble proteins. E18 retinal tissue was lysed in a buffer containing 2% proteomics grade Triton X-114 (VWR, 97063-868) and protease inhibitors (Thermo Fisher, A32955). The tissue was shaken for 1 h at 4°C to break up tissue, triturated with progressively finer needles (21G, 23G, and 27G) to generate a single-cell suspension, and then sonicated to disrupt membranes. After removal of nuclei and cell debris by centrifugation, the lysate was then cloud point extracted (see Bordier, 1981; Taguchi and Schatzl, 2014) followed by methanol precipitation.

Protein samples were resuspended in 2x Laemmli buffer containing 6M urea. Samples were diluted to 1x Laemmli buffer, and protein concentration was determined using the Pierce 660 assay (Thermo Fisher, 22662 and 22663). In total, 20–30μg of protein was loaded onto a 7.5% SDS-PAGE stain-free gel (Bio-Rad, 4568023). Separated proteins were transferred to an Immobilon-FL PVDF (Millipore, IPFL00010) membrane using a BioRad transblot semi-dry apparatus at 24V for 1 h with a buffer consisting of 48mM Tris pH 9.2, 39mM glycine, and 10% methanol. The blot was washed briefly with DPBS before blocking for 1 h with 1% casein in DPBS. After a brief wash, the blot was incubated overnight in primary antibody (Table 1) (1:1,000 Rb anti-ANO1 or 1:1,000 Rb anti-CFTR, 1% BSA, 0.01% thimerosal). It was washed with DPBS with 0.05% Tween 80, incubated for 1 h with goat anti-rabbit HRP secondary (1:100,000), and developed with Pierce SuperSignal West Pico PLUS (Thermo Fisher, 34580). The images were acquired on a BioRad ChemiDoc XRS+.

**Table 1:** Antibodies.

**Antibody**	**Supplier Cat# (RRID #)**
Rabbit anti-TMEM16A monoclonal	Abcam ab190803 (AB_2892592)
Rabbit anti-CFTR Polyclonal	Abcam ab131553 (AB_2893490)
Goat anti-Rabbit Alexa 488	ThermoFisher A-11034 (AB_2576217)
Goat anti-Rabbit Alexa 555	ThermoFisher A-21429 (AB_2535850)
Goat anti-Rabbit HRP	ThermoFisher A-16110 (AB_2534782)

### Preparation of Custom sgRNA and *in vitro* Validation

To identify candidate guide RNAs capable of targeting TMEM16A, we used the CRISPR 10K track in the UCSC Genome Browser and the Integrated DNA Technologies gRNA checker tool. The selected candidate gRNAs (Table 2) were then tested *in vitro* prior to the transfection of retinal neurons to ensure their on-target functionality. Transcription template oligos specific to *TMEM16A* were ordered from IDT, and transcription was performed according to the EnGen sgRNA Synthesis Kit (NEB, E3322V). Target DNA was PCR-amplified from E18 chick retina genomic DNA. We then assessed each guide RNA for on-target efficiency in parallel *in vitro* reactions. RNPs were formed separately with AltR S.p. HiFi Cas9 V3 (Integrated DNA Technologies, 1081060) for each transcribed sgRNA. Reactions were assembled using 500fmol of pre-assembled RNP and 25fmol of target DNA in a total reaction volume of 10 μl.

**Table 2:** Oligonucleotide sequences from the work in this paper.

**Oligo**	**Nucleotide sequence (5^′^-3^′^)**	**Product size (bp)**
TMEM16A F	TCCCAGACATTCCCAAGGAC	286
TMEM16A R	CCTGCCCTTTACATGATGGC	
TMEM16B F	TTGGCCGACCTGGTCATTAT	327
TMEM16B R	GTGTGACAAAGCCGAACTGG	
TMEM16C F	TTGCCTGGTTGGGATGGTAT	301
TMEM16C R	ACAGCTCTCCGTCTTTTCCA	
TMEM16D F	ATCAGGTGCTCATGACCCAA	384
TMEM16D R	TTTTGCACAGTGATCCTGCC	
TMEM16E F	CACCAAGCCCTTACCCTGTA	354
TMEM16E R	GAGCAAACTCTACTGCAGGC	
TMEM16F F	CATTTTCCCCCTGGTTTGGG	468
TMEM16F R	GTGACAGCTGCAAGAAACCA	
TMEM16H F	CAAAGCCTGGATGAAGACGG	510
TMEM16H R	GACTTCATCAGCCGCTTGAG	
TMEM16J F	GGTTCACCATCAAGAAAATTGAGGA	415
TMEM16J R	GCGAATCCCAGCAAGTAGGT	
TMEM16K F	CCCTACGTATGCCAGTTTGC	530
TMEM16K R	GTTCAAGGCCCAAGGAAGTG	
TMEM16A gDNA 434 F	GCCCATTTGACTGTGCACTAA	
TMEM16A gDNA 434 R	CACCAGCCATCGTCCTTATCAT	
TMEM16A gDNA 606 F	CCAGCTATCAGGAAAATTGC	
TMEM16A gDNA 606 R	TGTGATCTCCCCAAATCTAC	
TMEM16A gRNA 192	GAGGAAAGTGGATTACATCC	
TMEM16A gRNA 222	ACTACAAGAAGTCTTCAGCA	
TMEM16A gRNA 424	CCTGGAACTGGAACATGATG	
HiFiCas9_SDM_r691a_F	CTTCGCCAACGCGAACTTCATGCAGC	Vakulskas et al. (2018)
HiFiCas9_SDM_r691a_R	CCGTCGGACTTCAGGAAA	

### Ribonucleoprotein and Nucleofection

To genetically disrupt TMEM16A expression, tracrRNA, tagged with ATTO 550, and *in vitro* validated crRNAs (Integrated DNA Technologies) were annealed in a preheated thermocycler at 95°C for 5 min followed by cooling to 25°C at rate of −0.2°C/s. RNP complexes (120pmol gRNA, 104pmol Cas9, and 5 μl total) were formed in the Cas9 dilution buffer. RNPs, electroporation enhancer (Integrated DNA Technologies, 1075915), and Nucleofector solution (Lonza, VPG-1002) were combined with 4–6 × 10^6^ primary retinal neurons and electroporated using the Nucleofector IIb device (Lonza, AAB-1001). Control reactions were performed in parallel by transfecting 10 μl of Cas9/tracrRNA only. For plasmid transfections, 1 μg of P222 or control vector was electroporated as described above.

### Electrophysiology

Retinal cultures maintained for 6–11 days (E15-E20) were used for electrophysiological recordings to assess functional consequences of TMEM16A pharmacological inhibition or genetic disruption. Dishes were mounted on the stage of an Olympus IX70 inverted microscope, and a 3M KCl agarose bridge connected the reference Ag/AgCl pellet electrode to the dish. Patch electrodes were pulled from thick-walled borosilicate glass (Sutter Instruments, BF150-86-10) by a P-97 micropipette puller (Sutter Instruments, P-97) with a 2.5 mm square box filament (Sutter Instruments, FB255B). Electrode tips were pulled to produce a short taper with resistance between 5 and 8 MΩ. For the T16Ainh-A01 (50 μM) experiment, the inhibitor was added to the internal solution before recordings from a 50mM stock in DMSO. During the experiment, a continuous supply of external solution was delivered through a pressurized eight-channel perfusion system (Automate Scientific, 01-18). In total, 50 μl of bubbled NO solution was injected into the perfusion line using a gas-tight syringe attached to a repeating dispenser (Hamilton, 81220 and 83700). Whole-cell patch recordings were performed using an Axopatch 1D amplifier with pClamp 10.0 for inhibitor experiments. Recordings from transfected cells were performed with an Axopatch 200B amplifier and pClamp 11.2 software (Molecular Devices, Axopatch 200B-2). The voltage was held at −70 mV for 150 ms and stepped to −90 mV for 50ms, then changed from −90 to 50 mV over 200 ms, and returned to −70 mV. The first ramp recording was used to measure leak currents, and the second ramp was measured in the presence of GABA (20 μM). Series resistance was recorded for each cell, and junction potential was estimated in the pClamp 10.0 calculator to be −13 mV. The reversal potential of GABA-gated current (*E*_*revGABA*_) was calculated by correcting for series resistance and junction potential and then subtracting the current from the first ramp from the current from the second ramp. The external solution comprised 116.7 mM NaCl, 5.3 mM KCl, 20 mM tetraethylammonium (TEA)-Cl, 3 mM CaCl_2_, 0.41 mM MgCl_2_, 10 mM HEPES, 5.6mM glucose, 300 nM TTX, and 50 μM LaCl_3_. The internal solution comprised 100 mM Cs-acetate, 10 mM CsCl, 0.1 mM CaCl_2_, 2 mM MgCl_2_, 10 mM HEPES, and 1.1 mM EGTA, supplemented with an ATP regeneration system which comprised 1mM ATP disodium, 3 mM ATP dipotassium, 2 mM GTP disodium, 20 mM creatine phosphate, and 50U/ml creatine phosphokinase. All recordings were conducted at room temperature.

### Imaging Analysis and Statistics

For knockdown analysis, the images were collected with an Olympus IX-70 with 100x 1.35NA oil immersion objective using Slidebook software. Cells expressing the fluorescent reporter were considered transfected and were subsequently quantified for immunofluorescence of TMEM16A. Transfection with the all-in-one plasmid results in co-expression of tdTomato reporter with sgRNA/Cas9. Transfection of the pre-formed ribonucleoprotein with separate GFP plasmid means reporter expression does not guarantee successful RNP delivery. Cells were selected for tdTomato or GFP expression without prior knowledge of TMEM16A labeling to reduce bias. Analysis was done in Image J using auto local thresholding (Bernsen) to segment the image and create a binary mask for quantification of imaging data. Cells not expressing the fluorescent marker and all cell bodies were manually removed from the binary image so that only processes of transfected cells were analyzed. The D'Agostino–Pearson normality test was performed on samples, and any non-normal data were log-transformed prior to parametric statistical testing. Differences between groups were assessed using two-tailed Welch's *t-tests*. All statistics were performed with GraphPad Prism software, and figures were assembled in Adobe Illustrator.

## Results

**Figure 1:**
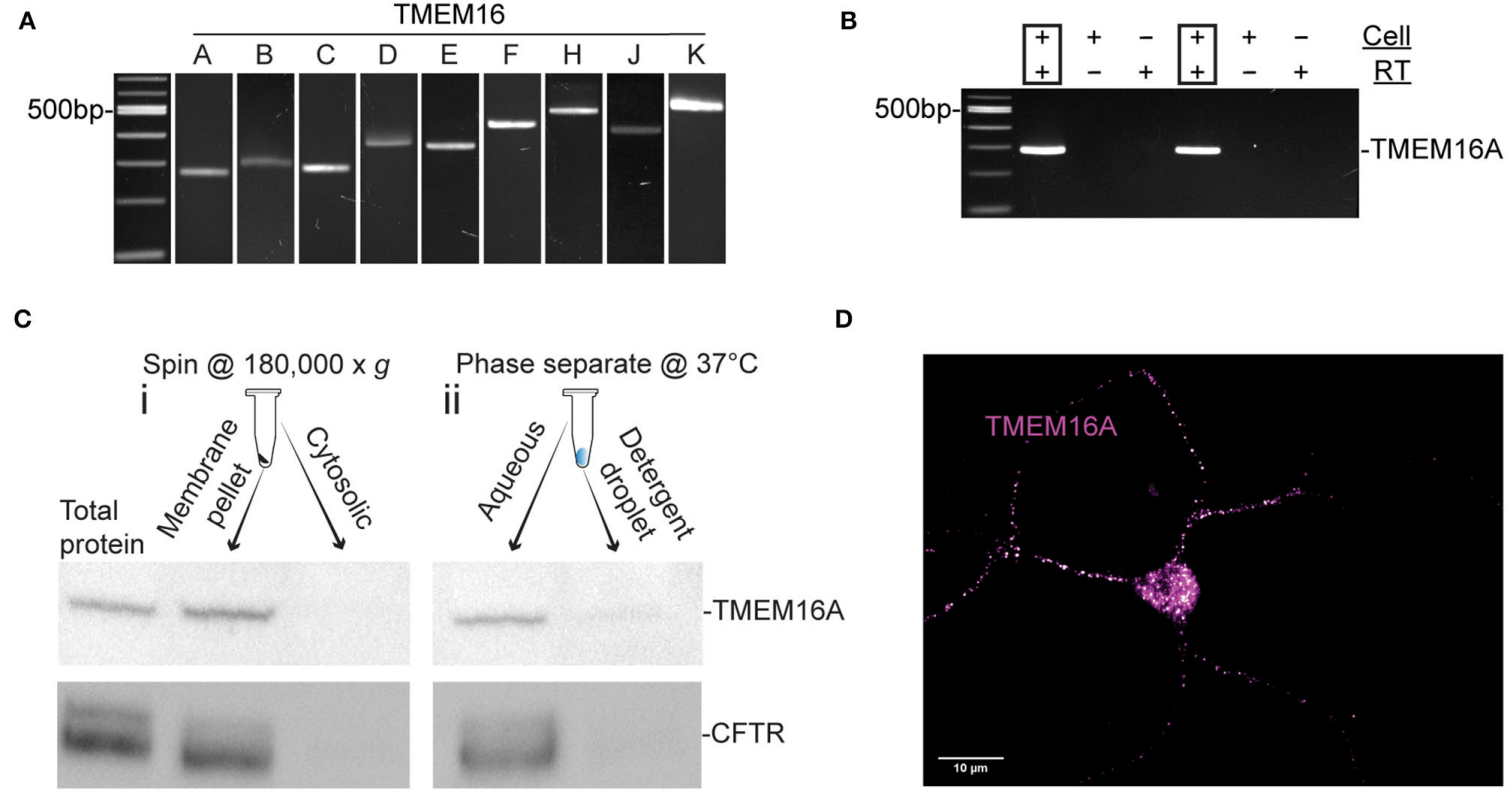
TMEM16A transcript and protein are expressed in retinal amacrine cells. **(A)** RT-PCR of RNA extracted from E16 retinal tissue for TMEM16 paralogues. **(B)** Single-cell PCR amplifying TMEM16A from individual AC. Each cell was DNase-treated prior to reverse transcription. RT (–) and template (–) controls showed no amplification. **(C)** Western blot of subcellular fractionated protein samples (i) and Triton x-114 cloud point extraction samples (ii) show immunodetection of TMEM16A (Abcam ab190803) and CFTR (Abcam ab131553) in the same fraction with both types of membrane preparation. **(D)** Immunocytochemistry for TMEM16A reveals a punctate labeling along with the soma. The image is displayed with a magenta-hot look-up table that shows puncta with high-fluorescence intensity as white.

Nitric oxide induces a cytosolic Cl^−^ elevation in ACs by the release of Cl^−^ from an internal acidic store. In addition, Ca^2+^ elevations were demonstrated (Maddox and Gleason, 2017; Zhong and Gleason, 2021) to activate adenylate cyclase 1, cAMP production, and stimulation of PKA which activates CFTR (Zhong and Gleason, 2021). Nonetheless, the full role of these Ca^2+^ elevations is not known. To investigate the possibility that a Ca^2+^-activated Cl^−^ channel is involved, we tested for expression of TMEM16 paralogs by RT-PCR in chick retinal cultures. Embryonic day 15-16 mixed chick retinal cultures expressed the transcripts TMEM16A, TMEM16B, TMEM16C, TMEM16D, TMEM16E, TMEM16F, TMEM16H, TMEM16J, and TMEM16K (Figure 1A). All products were sequence-verified. To determine whether TMEM16A is expressed in ACs specifically, single ACs were aspirated from the culture dish and tested *via* RT-PCR for TMEM16A transcript (Figure 1B). Because not all transcripts are translated into protein, Western blots were performed on extracts from E18 chicken retina to test for TMEM16A protein. Immunoblotting yielded a single band for TMEM16A at the expected molecular weight of ~117KDa (Figure 1C).

To assess the possibility of TMEM16A and CFTR interactions, cytosolic proteins were separated from membrane proteins and immunoblots of each fraction were performed for TMEM16A and CFTR. The membrane fraction generated by ultracentrifugation (Figure 1Ci) contained a heterogeneous mixture of membranes from the plasma membrane, mitochondria, endoplasmic reticulum, Golgi proteins, and other subcellular organelles. This assay showed both TMEM16A and CFTR in the membrane fraction while excluding both from the cytosolic fraction, as expected. However, Triton X-114 cloud point extraction unexpectedly enriched both proteins in the aqueous phase and excluded them from the detergent phase (Figure 1Cii). Hydrophobic transmembrane proteins are expected to partition to the detergent phase owing to their favorable interaction with non-ionic detergents. Such anomalous partitioning by Triton X-114 was reported for channel-forming membrane proteins possessing multiple transmembrane domains (Maher and Singer, 1985). Importantly, both proteins were found in the same fraction/phase by either method. The same TMEM16A antibody used for Western blots of mixed retinal cells also labeled ACs, specifically (Figure 1D). The representative fluorescent image uses a magenta-hot look-up table that displays puncta of increasing fluorescent intensity from magenta to white, respectively. Labeling appeared as distinct puncta in the soma and processes of ACs in culture (Figure 1D). It was commonly observed that amacrine cells have one–two processes with higher density labeling compared to other processes of the same cell.

**Figure 2:**
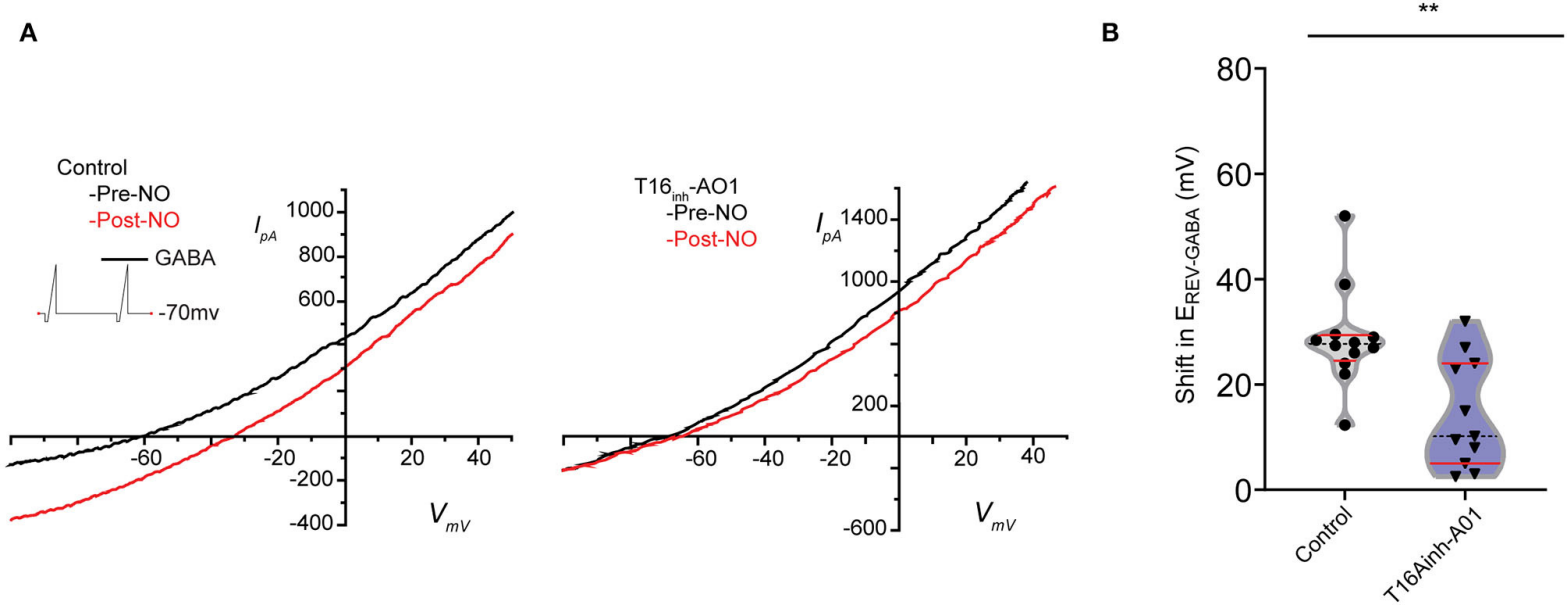
Pharmacological inhibition of TMEM16A reduces the transient shift in *E*_*revGABA*_. **(A)** Current–voltage relationship for leak subtracted GABA-gated currents recorded before and after NO injection in control and TMEM16A inhibitor (T16_inh_-AO1) conditions. **(B)** Truncated violin plots for the mean shift in the reversal potential of GABA-gated currents *(E*_*revGABA*_). The shape of the violin shows frequency distribution of the data, top and bottom of the violin are data extrema, red lines are quartiles, and the dashed line is the median of the data. The mean shift in *E*_*revGABA*_ was reduced, and frequency distribution becomes bimodal when TMEM16A inhibitor (*n* = 11) was in the external solution compared to control conditions (*n* = 12). Recordings were from E16 cultures (Welch's *t*-test, ** denotes *p* < 0.005).

To investigate whether TMEM16A contributes to the NO-dependent elevation of cytosolic Cl^−^, we monitored the reversal potential of the Cl^−^ current through open GABA_A_ receptors (*E*_*revGABA*_) before and after NO (Figure 2A). Pairs of voltage ramps were delivered to ACs recorded in the voltage-clamp configuration. The first ramp was delivered without GABA, and the second ramp was delivered in the presence of 20 μM GABA to establish *E*_*revGABA*_ without NO. A subsequent double-ramp recording was made on the same cell after exposure to NO. The difference in *E*_*revGABA*_ measured from the recordings before and after NO constitutes the shift in *E*_*revGABA*_ due to the NO-dependent Cl^−^ release. To test the involvement of TMEM16A in the NO-dependent Cl^−^ release, we examined the effect of including the TMEM16A/TMEM16B inhibitor T16_inh_-A01 in the recording pipette. Under control conditions in the absence of an inhibitor, the NO-induced shift in *E*_*revGABA*_ (28.7 ± 2.8 mV, *n* = 12) was significantly higher than the shift from ACs recorded with T16_inh_-A01 in the pipette (14.5 ± 3.1 mV, *n* = 11, Welch's *t*-test *p* < 0.005, Figure 2B). These results suggest that TMEM16A/B is involved in the NO-dependent Cl^−^ release of retinal ACs.

Pharmacological inhibitors for Cl^−^ channels are notoriously promiscuous (Sepela and Sack, 2018). Therefore, we sought to evaluate the involvement of TMEM16A more specifically by employing CRISPR/Cas9. CRISPR/Cas9 produces double-stranded breaks in the targeted genetic locus that are erroneously repaired by non-homologous end-joining to yield insertions and deletions (indels). ACs do not divide in culture to produce clones meaning these indels are heterogeneous among different ACs in culture.

**Figure 3:**
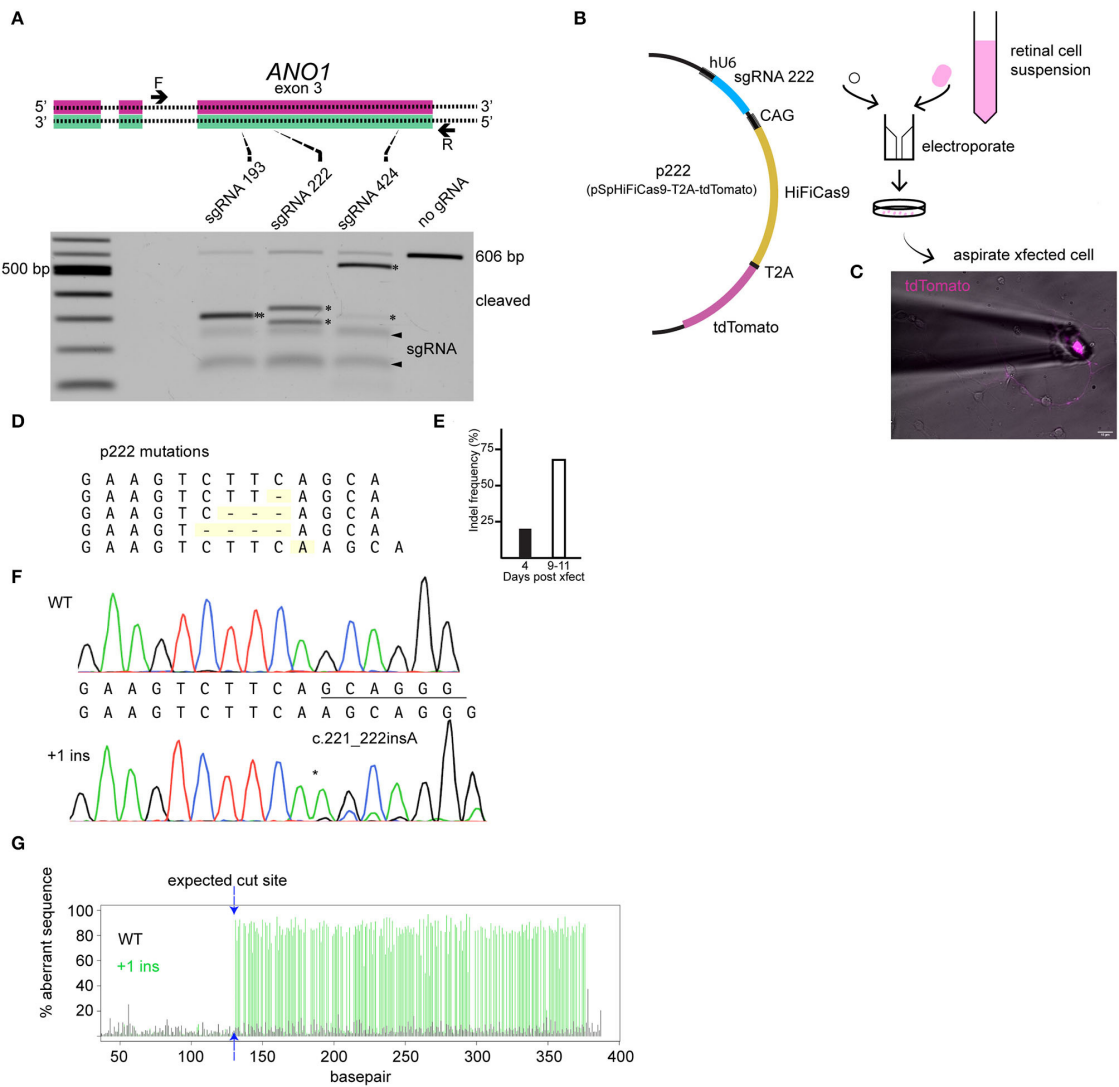
CRISPR/Cas9 edits *TMEM16A* of retinal amacrine cells when delivered by an all-in-one plasmid. **(A)** (top) Graphical representation of target genetic loci showing the cut site for each sgRNA. Primers are denoted by the arrows, and sgRNA target regions are indicated by the dashed lines. (Bottom) Agarose gel electrophoresis of *in vitro* Cas9 cleaved DNA (asterisks) for each sgRNA of a 606 bp PCR product containing exon 3 of *TMEM16A*. The pair of faint bands below the digestion fragments correspond to sgRNA secondary structures (arrowheads). **(B)** Simplified plasmid map of pSpHiFiCas9-T2A-tdTomato which was electroporated into dissociated chick retinal cells. **(C)** AC in culture expressing tdTomato is aspirated into a glass pipette for single-cell genomic PCR. **(D)** List of observed mutations detected in p222 transfected cells. **(E)** Percent indels detected from 4 days and 9–11 days post-transfection. **(F)** Representative sequence traces from genomic PCR of a wild-type cell (wt/wt) and an edited cell with ~30% WT and 70% +1 insertion alleles (wt/c221_222insA) according to TIDE. **(G)** TIDE output from cell in **(F)** shows substantial aberrant sequence starting at the expected cut site due to a mixture of WT and +1 amplicons in the edited cells PCR. * indicates cleavage products.

The editing tool was delivered on the day neurons are plated (embryonic day 9). TMEM16A protein may be present at the time of transfection; therefore, loss of TMEM16A function will only be evident once pre-existing TMEM16A protein is turned over. We designed three guide RNAs against exon 3 of the TMEM16A gene (*ANO1*) and tested them by *in vitro* Cas9 digestion to confirm effectiveness. The target region of the TMEM16A gene (exon 3 of *ANO1*) was amplified by PCR and incubated with each respective Cas9/sgRNA ribonucleoprotein. Gel electrophoresis of the digests confirmed the on-target efficacy of each sgRNA on the purified TMEM16A target (Figure 3A). Guide RNA 222 was chosen for further testing in mixed retinal cell cultures for its greater out-of-frame indel formation score (Bae et al., 2014) compared to the other two guide RNAs. Cells were transfected with a plasmid expressing HiFi Cas9 (Vakulskas et al., 2018), tdTomato, sgRNA 222 (p222), and control cells that were transfected with the same vector minus sgRNA 222 (Figure 3B). To assess editing events, individual ACs expressing tdTomato were collected and subjected to genomic amplification (Figure 3C). The target region of the TMEM16A gene (*ANO1*) was amplified using a set of primers to give a 434 bp product. The identity of the PCR product and the presence of indels were evaluated by Sanger sequencing. We detected a range of insertions and deletions with the most common being a 1 bp insertion (Figure 3D). Single ACs were tested for indels by genomic PCR at 4 days post-transfection and 9–11 days post-transfection which resulted in 20% (1/5) and 67% (10/15) cells with indel mutations per cell amplified, respectively (Figure 3E). Figure 3F shows a representative sequencing chromatogram for a wild-type cell and a cell heterozygous for a +1 insertion mutation. The adjacent TIDE analysis (Figure 3G) shows a high aberrant sequence after the edit, owing to a mixture of a +1 insertion and wild-type sequence. Altogether, these results confirm the efficacy of vector p222 to generate indel mutations in ACs.

**Figure 4:**
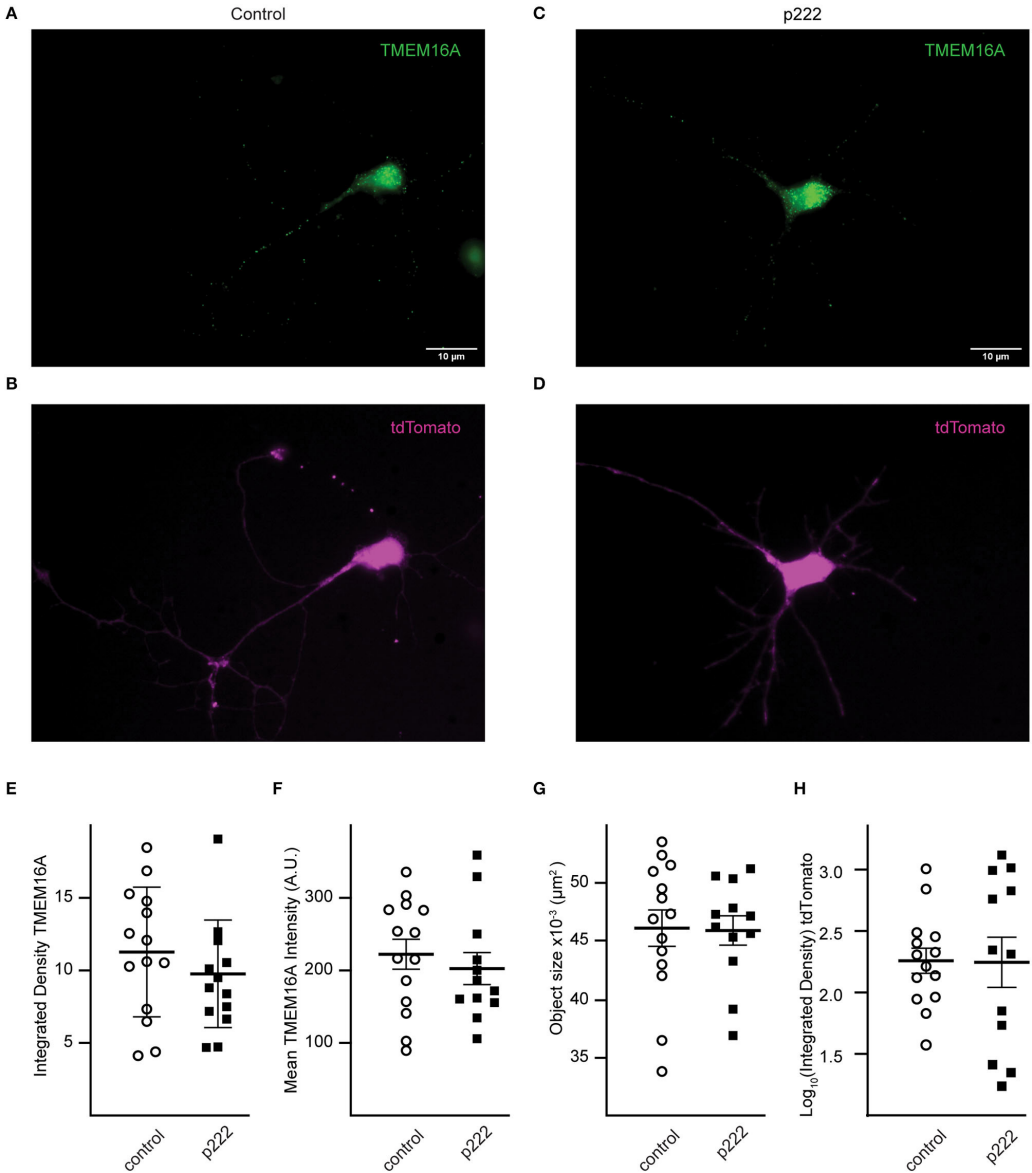
Transfection with P222 has no effect on TMEM16A protein expression **(A–D)**. Representative images of TMEM16A antibody immunofluorescence and tdTomato fluorescence from e18 cells transfected with control plasmid **(A,B)** or p222 plasmid **(C,D)** targeting TMEM16A. **(E)** The mean integrated density for all objects identified within the TMEM16A mask; **(F)** the mean fluorescent intensity of the objects; **(G)** the mean objects size; **(H)** the log-transformed integrated density for tdTomato. There is no statistical difference between groups in **(E–H)** (two-tailed Welch's *t-tests*).

To detect loss of TMEM16A protein, immunocytochemistry was performed using the anti-TMEM16A antibody on transfected cells. Representative cells from the analysis are shown in Figures 4A–D. There was no significant difference in mean integrated density (sum of raw pixel intensities in the fluorescent object) (control = 11.24 ± 1.19, p222 = 9.75 ± 1.06, Figure 4E), mean fluorescent intensity (control =222.40 ± 77.31, p222= 202.73 ± 76.09, Figure 4F), or the mean size of fluorescent objects immunoreactive for anti-TMEM16A antibody (control = 4.61 × 10^−2^ ± 1.56 × 10^−3^ μm^2^, p222 = 4.59 × 10^−2^ ± 1.17 × 10^−3^ μm^2^, Figure 4G). We compared the expression of tdTomato and TMEM16A to determine whether higher expression of the construct was associated with reduced TMEM16A. The log-transformed integrated density (Figure 4H) for tdTomato is not different between groups (*p* = 0.96) (control *n* = 14, p222 *n* = 12). Pearson's correlation coefficients were determined between tdTomato and TMEM16A to control for reporter expression effects on TMEM16A immunolabeling and showed minimal correlation (control *r* = 0.36 with *p* > 0.21, p222 *r* = 0.28 with *p* > 0.38) (control *n* = 14, p222 *n* = 12). From the data, we concluded that the knockdown was ineffective given the lack of difference in TMEM16A expression between groups.

**Figure 5:**
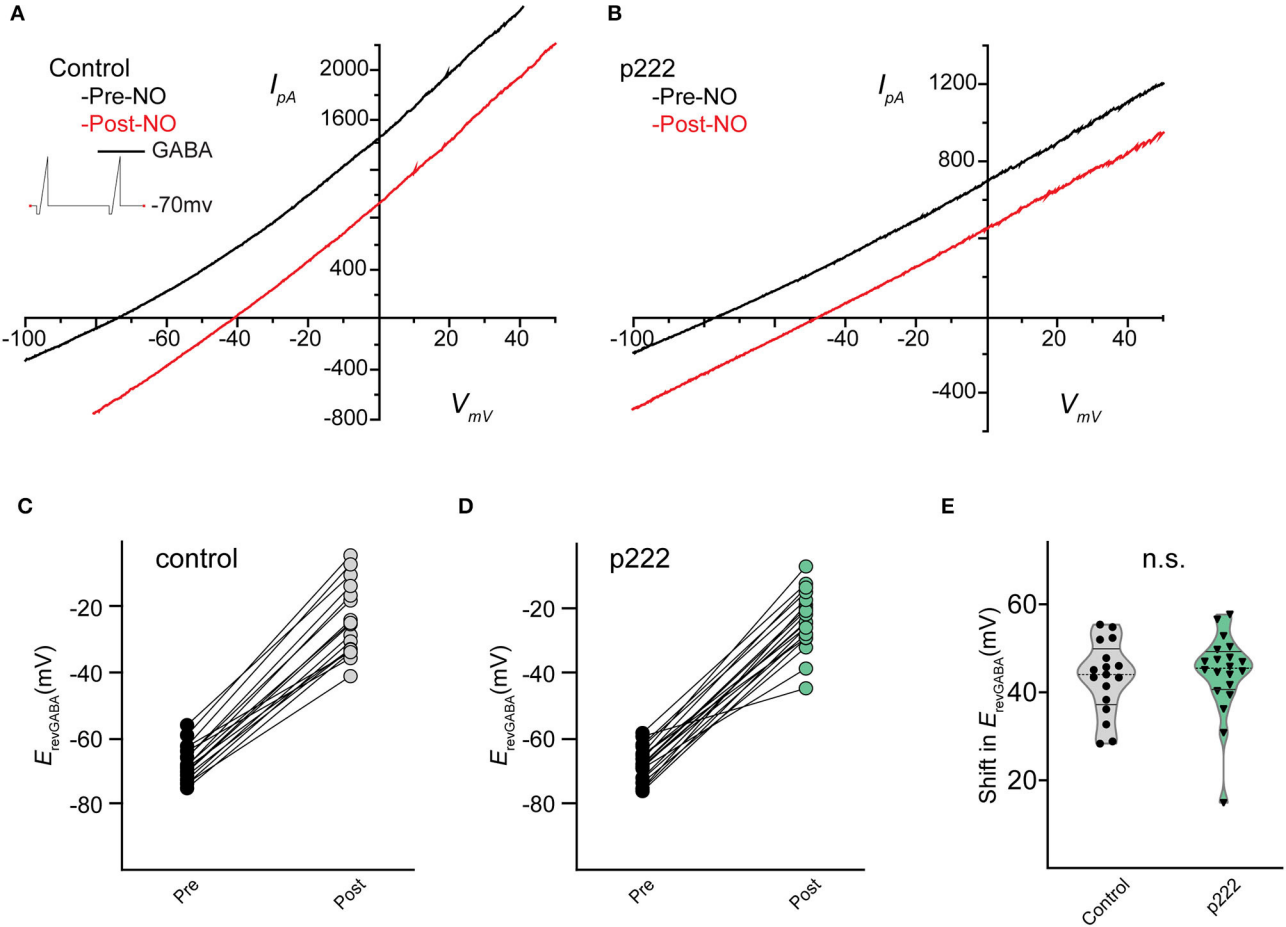
Mean shift in the reversal potential of GABA_A_Rs for control and cells targeted with p222 do not significantly differ. Current–voltage relationship for leak subtracted GABA-gated currents in a representative control AC **(A)** and in a representative p222 transfected cell **(B)**. Paired data points for *E*_*revGABA*_ before and after NO injection shift positively for all cells tested in control **(C)** and p222 transfected **(D)** groups. Violin plots of the mean shift in *E*_*revGABA*_ **(E)** show no difference and similar frequency distribution between control and p222 transfected groups (control *n* = 17, p222 *n* = 20, *p* = 0.75 two-tailed Welch's *t-tests*).

Whole-cell recordings in the voltage-clamp mode were performed on cells transfected by vector p222 to evaluate the effect of indel mutations on the NO-dependent shift in *E*_*revGABA*_ (Figure 5). A pair of voltage ramps were delivered with GABA applied during the second ramp of the pair. A subsequent recording was made on the same cell with a bolus of NO injected during the recording to initiate the shift (Figures 5C,D). No significant difference in the NO-dependent shift in *E*_*revGABA*_ was observed between cells targeted for TMEM16A (*ANO1*) by p222 and cells expressing the construct without the guide RNA (Figure 5E, control, 43.28 ± 2.012 mV vs. p222, 44.20 ± 2.096 mV, Welch's *t*-test) consistent with the immunocytochemistry.

**Figure 6:**
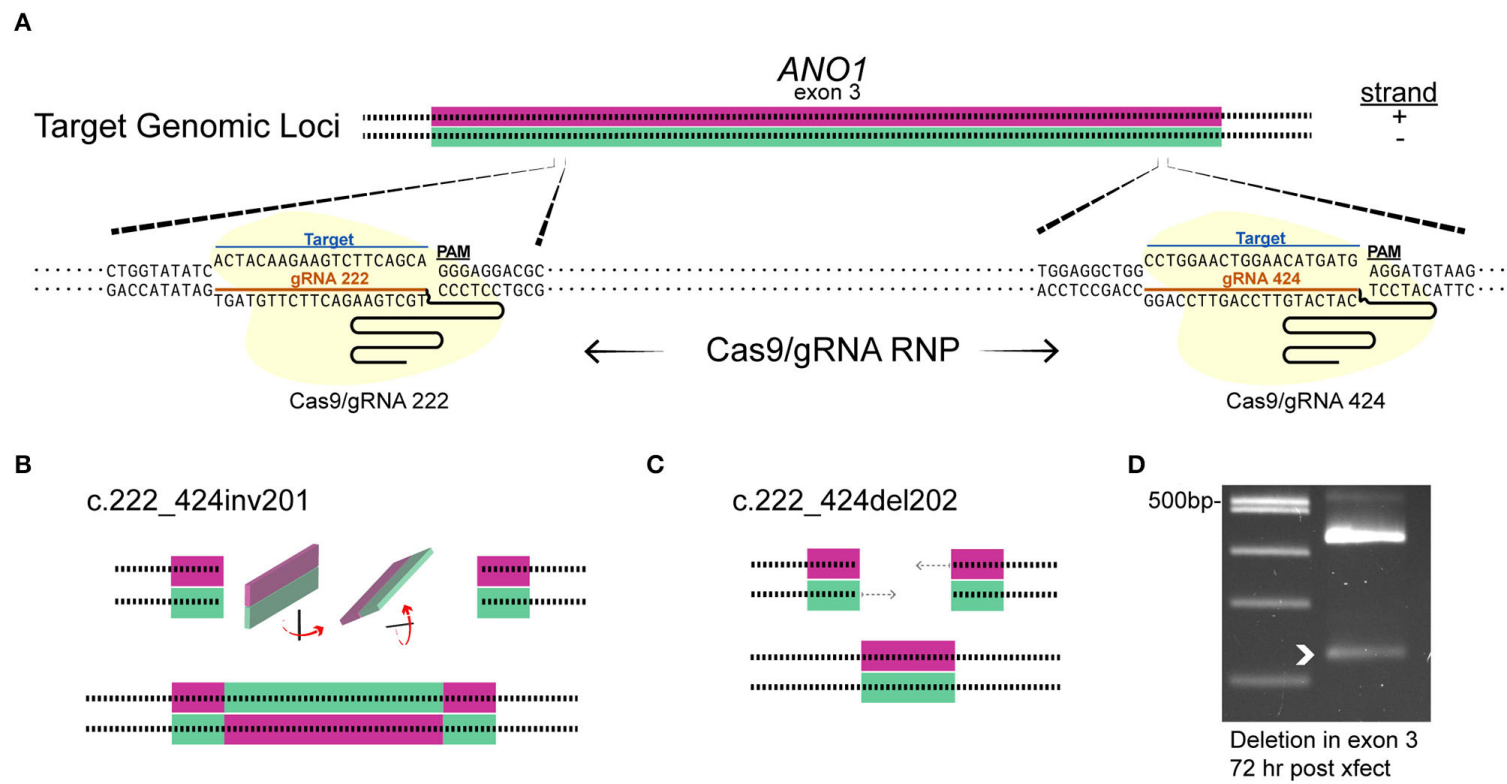
Targeting *TMEM16A* exon 3 with dual gRNA Cas9 ribonucleoprotein produces deletions within 3 days. **(A)** The target loci in *TMEM16A* showing cleavage sites. **(B,C)** Graphical depictions of observed mutations from single amacrine cells that were dual gRNA-transfected showing an inversion c.222_424inv201 **(B)** and deletion c.222_424del202 **(C)** between the two target sites. **(D)** Edits were present as a smaller PCR amplicon from a separate amplification of mixed cultures 3 days post-transfection (white arrowhead).

Since the plasmid delivery system takes >4 days for efficient indel mutagenesis in ACs (see Figure 3E) and the predicted turnover rate of TMEM16A could be days to weeks, (Bill et al., 2014), the pre-formed RNA/Cas9 ribonucleoprotein was used to target TMEM16A (*ANO1*). This method is expected to produce edits within 1–3 days before being degraded by the cell, thus shortening the timeframe to a detectable loss in protein. To increase the likelihood of editing events affecting TMEM16A protein expression in our experimental window (7–11 days post-transfection), we simultaneously delivered Cas9 RNPs formed separately with two guide RNAs (gRNAs 222 and 424) targeting exon 3 of TMEM16A (Figure 6A). Single-cell genomic PCR revealed an inversion mutant and deletion mutant for the portion of exon 3 between the two guide RNAs (Figures 6B,C). Genomic PCR from pooled lysates also showed a deletion within exon 3 of TMEM16A (*ANO1*) detectable 72 h after transfection of cultured mixed retinal cells (Figure 6D).

**Figure 7:**
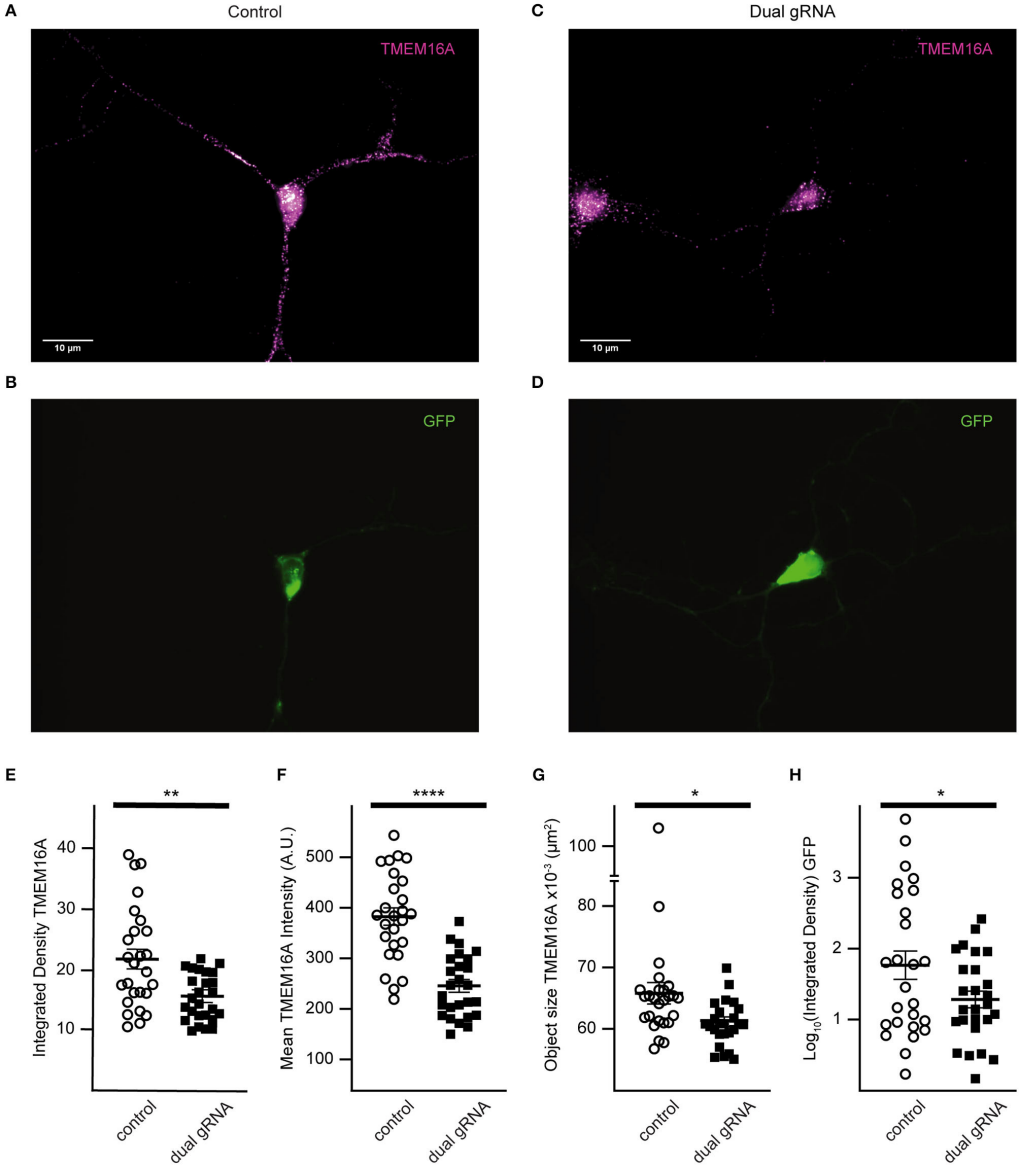
Dual gRNA Cas9 ribonucleoprotein reduces *TMEM16A* expression **(A–D)**. Representative immunofluorescence images of GFP expressing amacrine cells labeled with TMEM16A antibodies in control-transfected **(A,B)** and dual-guide RNA-transfected **(C,D)** conditions. **(E)** The mean integrated density for all objects identified within the TMEM16A mask is reduced in the dual-guide condition. **(F)** The mean fluorescent intensity of TMEM16A objects and the mean TMEM16A object size **(G)** are significantly reduced in dual-guide RNA-transfected cells. **(H)** Log-transformed integrated density of the GFP signal is lower in dual gRNA group. * denotes *p* < 0.05, ** denotes *p* < 0.005, **** denotes *p* < 0.00005.

TMEM16A protein expression of RNP targeted cells was examined by immunofluorescence (Figures 7A–D). Quantitative analysis was done only for cells co-expressing GFP to reduce selection bias. The mean integrated density for all objects identified within the TMEM16A mask is reduced in the dual-guide condition (control = 21.8 ± 1.64, dual gRNA = 15.5 ± 0.76, *p* = 0.0014) (Figure 7E). The mean fluorescent intensity of the objects (control = 382.8 ± 17.26, dual gRNA = 244.5 ± 11.96, *p* = 4.37 × 10^−8^) (Figure 7F) and the mean object size (control = 6.58 × 10^−2^ ± 1.74 × 10^−3^ μm^2^, dual gRNA = 6.10 × 10^−2^ ± 7.28 × 10^−4^ μm^2^, *p* = 0.017) (Figure 7G) are significantly reduced as well. These results suggest a loss of TMEM16A protein when transfected with the dual-guide RNP. The log-transformed integrated density for GFP expression is lower in the dual gRNA group (control = 1.81 ± 0.19, dual gRNA = 1.36 ± 0.11, *p* = 0.042) (Figure 7H); however, there is no significant correlation between GFP expression and TMEM16A immunolabeling for control (r = 0.074 with *p* > 0.71) or dual gRNA/RNP transfection (*r* = 0.19 with *p* > 0.34) (Figures 7E,F) (control n = 26, dual gRNA/RNP *n* = 26), which suggests that GFP expression is independent of TMEM16A detection in the immunocytochemistry analysis.

**Figure 8:**
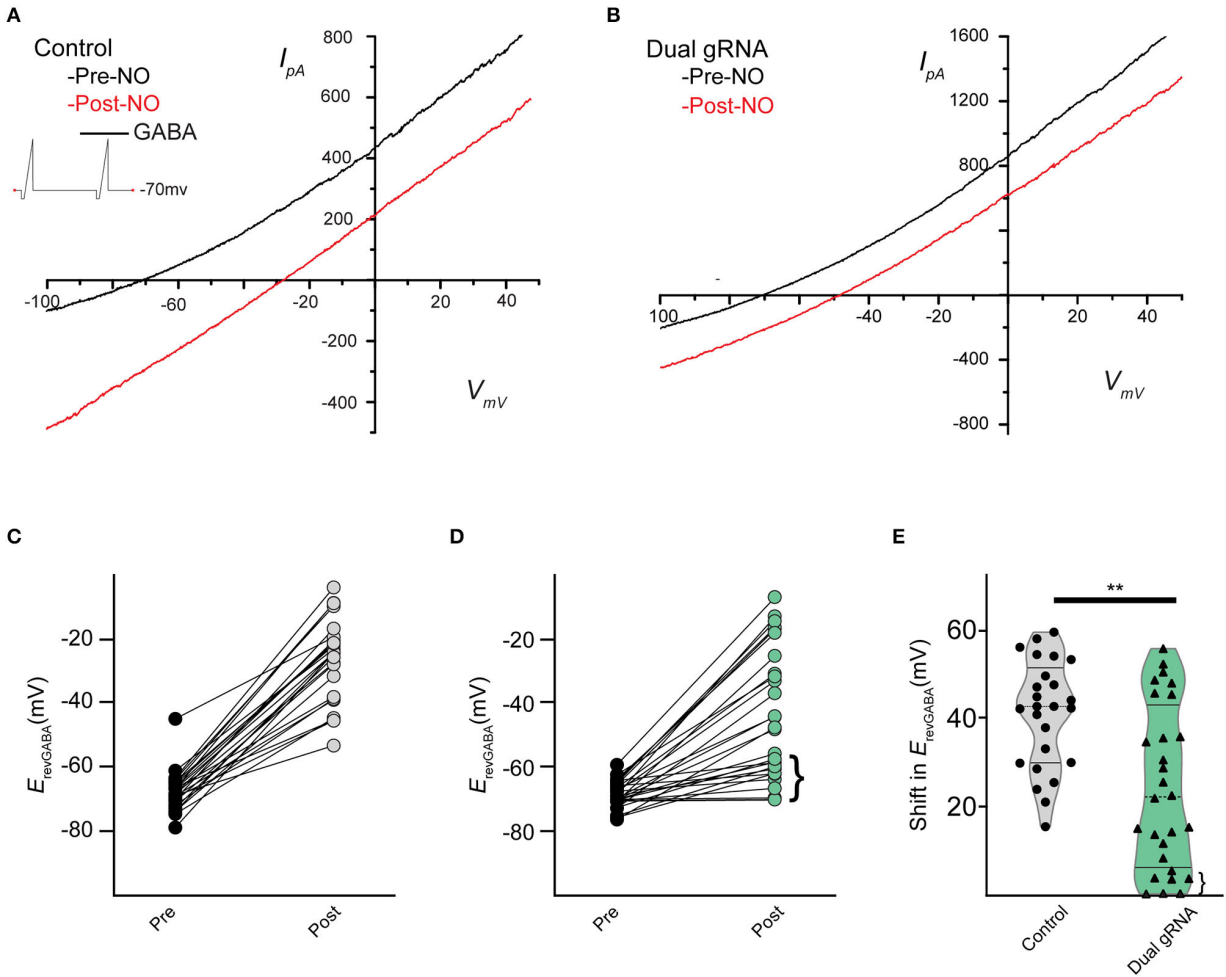
Dual gRNA Cas9 ribonucleoproteins targeting *TMEM16A* reduce the NO-dependent shift in *E*_*revGABA*_. Recording traces from GABA-gated currents before and after injecting NO from control-transfected **(A)** (*n* = 23) and dual gRNA/RNP-transfected cells **(B)** (*n* = 25). Paired data showing before and after NO injection for control **(C)** and dual-guide RNA targeting TMEM16A **(D)**. Delivery of the Cas9 ribonucleoprotein led to a population of cells exhibiting significantly reduced NO-dependent shift [**(D)**, brace] **(E)** Violin plots for the mean shift in *E*_*revGABA*_ show a reduction under dual gRNA/RNP condition and a lower quartile [**(E)**, brace] that is far lower than control (control *n* = 25, dual gRNA/RNP *n* = 28, *p* = 0.003, ** denotes *p* < 0.005).

Recording from GFP expressing cells, we assessed the NO-dependent release of Cl^−^ in RNP-transfected cells and saw a significant reduction in the NO response (Figures 8A,B). Under control conditions, NO induced a mean shift in *E*_*revGABA*_ of 39.6 ± 2.4 mV (*n* = 23) (Figure 8C), but in the RNP group targeting TMEM16A, the mean NO-induced shift in *E*_*revGABA*_ was 23.8 ± 3.7 mV (Figure 8D) (*n* = 25, p = 0.001 unpaired *t*-test). Notably, there were a group of three cells (Figure 8E, pink) with a shift of <1 mV which may correspond to the ACs lacking any TMEM16A expression. In addition, the lower quartile (Figures 8D,E, braces) may represent a subset of ACs that require full TMEM16A expression for Cl^−^ transport in response to nitric oxide signaling, whereas other subsets do not.

## Discussion

Here, we show that chick retinal ACs express the TMEM16A transcript and protein. Pharmacological inhibition of TMEM16A leads to inhibition of the NO-dependent release of internal Cl^−^ and the shift in *E*_*revGABA*_. This suggests that TMEM16A plays a role in the modulation of intracellular Cl^−^ in retinal ACs. To confirm the results with T16inh-AO1 inhibition, we utilized CRISPR/Cas9 in chick retinal ACs to specifically knock down TMEM16A. Since we use non-dividing cell cultures, mutations are heterogeneous and TMEM16A protein may be present before genetic disruption by CRISPR/Cas9. While an all-in-one plasmid encoding the sgRNA and HiFi Cas9 led to indel formation, it neither resulted in a significant loss of TMEM16A protein nor a detectable change in the NO-dependent Cl^−^ release over the time frame of culture viability. The plasmid takes days for its components to be transcribed and translated, and for Cas9 to combine with the guide RNA in the nucleus for targeted cleavage and erroneous repair (Callif et al., 2017). After an indel is formed, TMEM16A knockdown depends on its rate of degradation which may differ among cell types. Vascular tissue with lower expression of TMEM16A was unaffected by a constitutively expressed *Tmem16a-*siRNA compared to tissue with higher expression in a previous murine study (Jensen et al., 2018). TMEM16A was shown to contribute significantly toward Ca^2+^-dependent Cl^−^ secretion even when expressed at low levels in murine airway epithelia (Scudieri et al., 2012). In a separate study, inducible knockout of TMEM16A (*ANO1*) in mouse interstitial cells of Cajal resulted in protein loss that was patchy and incomplete even 2 months after induction (Malysz et al., 2017). In Te11 and FaDu cells, the half-life of TMEM16A was >24 h as determined by cycloheximide inhibition of new protein synthesis (Bill et al., 2014). This suggests that TMEM16A can have a relatively long half-life, especially when expressed at low levels yet still contributes significant Ca^2+−^dependent Cl^−^ conductance. The time it takes for the plasmid to form indels may have exceeded our experimental window (7–11 days post-transfection) when considering TMEM16A protein stability. This contrasts with our previous work with CFTR that utilized an all-in-one plasmid encoding the CFTR, targeting sgRNA and wtCas9. This method did show a loss of protein and loss of the NO-dependent Cl^−^ release. Importantly, CFTR is subjected to rapid degradation immediately following protein synthesis. Intracellular precursor forms of CFTR are reported to have a half-life of ~33min (Ward and Kopito, 1994).

When the CRISPR/Cas9 RNP was used, there was a significant change in protein expression and a reduction in NO-dependent Cl^−^ release 7–10 days post-delivery. Since the GFP-encoding plasmid is co-transfected with the dual gRNA/RNP, GFP expression does not guarantee successful RNP delivery. Thus, we likely underestimated the effect of knockdown and knockout because some unknown proportion of cells in the test group expressed GFP yet did not receive the RNP. Still, a few cells appeared to have TMEM16A knocked out entirely after RNP transfection. These cells had no appreciable immunoreactivity for the anti-TMEM16A antibody as determined by immunocytochemistry. Such complete absence of TMEM16A immunoreactivity was never observed in control or plasmid transfected conditions. Also, the frequency of complete knockout corresponds to a similar proportion of cells that have a loss of NO-dependent Cl^−^ release. Because the knockout of TMEM16A in intestinal epithelia expressing wild-type CFTR eliminated cAMP-activated Cl^−^ currents (Benedetto et al., 2017), it seems plausible that CFTR is responsible for the NO-dependent Cl^−^ release but requires expression of the TMEM16A to function in at least a subset of AC cell types. TMEM16A may also work in parallel to provide a direct link between Ca^2+^ and Cl^−^ conductance on a faster time scale than AdC1/cAMP-mediated Cl^−^ release *via* CFTR (Zhong and Gleason, 2021).

We identify the expression of TMEM16A transcript and protein in chick retinal ACs, in line with what has been observed in mouse retina (Jeon et al., 2013). Ca^2+^-activated Cl^−^ currents (CaCCs) mediated by TMEM16A are found in mouse rod bipolar cells (Jeon et al., 2013; Paik et al., 2020) and probably mediate the CaCCs identified in goldfish bipolar cells (Okada et al., 1995). However, no CaCC was found in chick ACs (Gleason et al., 1994), suggesting an intracellular localization for TMEM16A. Schreiber et al. show that TMEM16A localization may be cell- and tissue-specific depending on the expression of other TMEM16 paralogues that affect trafficking and function (Schreiber et al., 2010, 2015). In our study, we found mRNA transcripts for TMEM16 paralogues B, C, D, E, F, H, J, and K in embryonic chick retinal tissue. TMEM16G is absent from the *Gallus gallus* reference genome assembly (GRCg6a). The medley of TMEM16 paralogues expressed by individual ACs is not yet known, and AC subtypes may differ in TMEM16 expression profiles. Defining the compliment of TMEM16 paralogue expression in single cells could facilitate AC subtype identification in culture.

In our original paper on the NO-dependent release of internal Cl^−^ (Hoffpauir et al., 2006), we proposed a simple scenario where, in the presence of NO, ACs receiving input from bipolar cells and other amacrine cells would experience a shift in the balance of inputs such that ACs would be more depolarized and thus release more GABA or glycine onto postsynaptic cells and alter the retinal output. AC subset-specific co-expression of specific compliments of TMEM16 with CFTR could adjust the sensitivity of ACs to NO and fine-tune the NO response. Neuromodulation by the NO-dependent release of internal Cl^−^ further enhances AC flexibility by allowing a single neurotransmitter to provide both inhibitory and excitatory signals. In this way, circuit flexibility is maximized while metabolic, genetic, and spatial costs are minimized (Grimes et al., 2010) in agreement with the laws of the economy of space, time, and matter (y Cajal, 1999; Chklovskii, 2004).

## Data Availability Statement

The raw data supporting the conclusions to this article will be made available by the authors upon request.

## Ethics Statement

The animal study was reviewed and approved by LSU Institutional Animal Care and Use Committee.

## Author Contributions

TR and EG conceived and designed experiments, interpreted results, and prepared figures and drafted manuscript. TR, LZ, and HS performed experiments. All authors contributed to the article and approved the submitted version.

## Conflict of Interest

The authors declare that the research was conducted in the absence of any commercial or financial relationships that could be construed as a potential conflict of interest.

## Publisher's Note

All claims expressed in this article are solely those of the authors and do not necessarily represent those of their affiliated organizations, or those of the publisher, the editors and the reviewers. Any product that may be evaluated in this article, or claim that may be made by its manufacturer, is not guaranteed or endorsed by the publisher.
